# Successful implementation of Helping Babies Survive and Helping Mothers Survive programs—An Utstein formula for newborn and maternal survival

**DOI:** 10.1371/journal.pone.0178073

**Published:** 2017-06-07

**Authors:** Hege L. Ersdal, Nalini Singhal, Georgina Msemo, Ashish KC, Santorino Data, Nester T. Moyo, Cherrie L. Evans, Jeffrey Smith, Jeffrey M. Perlman, Susan Niermeyer, on behalf of the participants in the Utstein consensus process: How to implement successful Helping Babies Survive and Helping Mothers Survive programs

**Affiliations:** 1 Department of Anaesthesiology and Intensive Care, Stavanger University Hospital, Stavanger, Norway; 2 Division of Neonatology, University of Calgary, Calgary, Canada; 3 Reproductive and Child Health Services, Ministry of Health and Social Services, Dar es Salaam, Tanzania; 4 Department of Women’s and Children’s Health, Uppsala University, Uppsala, Sweden; 5 Health Section, UNICEF, Kathmandu, Nepal; 6 Pediatrics and Child Health, Mbarara University of Science and Technology, Mbarara, Uganda; 7 International Confederation of Midwives, Harare, Zimbabwe; 8 Jhpiego, John Hopkins University, Baltimore, Maryland, United States of America; 9 Division of Newborn Medicine, Weill Cornell Medical College, New York, New York, United States of America; 10 Section of Neonatology, University of Colorado School of Medicine, Colorado, Aurora, Colorado, United States of America; Centre Hospitalier Universitaire Vaudois, FRANCE

## Abstract

Globally, the burden of deaths and illness is still unacceptably high at the day of birth. Annually, approximately 300.000 women die related to childbirth, 2.7 million babies die within their first month of life, and 2.6 million babies are stillborn. Many of these fatalities could be avoided by basic, but prompt care, if birth attendants around the world had the necessary skills and competencies to manage life-threatening complications around the time of birth. Thus, the innovative Helping Babies Survive (HBS) and Helping Mothers Survive (HMS) programs emerged to meet the need for more practical, low-cost, and low-tech simulation-based training. This paper provides users of HBS and HMS programs a 10-point list of key implementation steps to create sustained impact, leading to increased survival of mothers and babies. The list evolved through an Utstein consensus process, involving a broad spectrum of international experts within the field, and can be used as a means to guide processes in low-resourced countries. Successful implementation of HBS and HMS training programs require country-led commitment, readiness, and follow-up to create local accountability and ownership. Each country has to identify its own gaps and define realistic service delivery standards and patient outcome goals depending on available financial resources for dissemination and sustainment.

## 1. Introduction

In 2013, almost 300.000 maternal and 2,7 million newborn deaths occurred globally [1,2]. Stillbirths accounted for another 2,6 million deaths yearly [3]. Since 2003 the maternal mortality ratio has been gradually decreasing, with a global annual decline of 2.7% [1]. However, there is a substantial variation in number and cause of maternal deaths between countries, with the highest burden in Africa and the Middle East, where most deaths are secondary to intrapartum or postpartum complications [1]. In 2013, almost 42% of global under-5 child mortality occurred in the neonatal period, reflecting the progressive increase in the proportion of neonatal deaths relative to other causes since the 1970s [2]. The global annual rate of decline in early neonatal deaths has been 1.2–1.4%, slower than for the overall under-5 mortality [2]. A similar slow rate of reduction is also estimated for intrapartum-related stillbirths [3], and a substantial number of stillbirths and neonatal deaths are caused by intrapartum-related complications, prematurity or small size at birth, and infection.

Although there have been accelerated reductions since the Millennium Declaration in 2000, global rates of change in many countries did not meet the Millennium Development Goal (MDG) 4 and 5 by 2015 [1,2]. The Sustainable Development Goal (SDG) 3 relating to health includes a two-thirds reduction in maternal mortality and ending preventable newborn deaths by 2030 [4]. To reach these targets, careful analysis and corresponding interventions are needed within different countries, especially in regions with slow progress.

### 1.1 Helping babies survive and helping mothers survive programs

Each year approximately 136 million babies are born, and as many as 15% of these births will encounter potentially life-threatening complications [5,6]. The concept of emergency obstetric and newborn care (EmONC) was introduced in 1997 by WHO, UNICEF and UNFPA to accelerate worldwide delivery of evidence-based services to reduce morbidity and mortality related to birth. Universal access to EmONC is considered essential to reduce maternal and neonatal mortality [6].

Unfortunately, the impact on patient outcomes resulting from many different training initiatives in low-resource countries over the past decades has been limited [7]. Thus, the innovative Helping Babies Survive (HBS) and Helping Mothers Survive (HMS) programs emerged to meet the need for more practical, basic, low-cost, low-tech simulation-based training. The first module in the HBS series, Helping Babies Breathe (HBB), was developed under the leadership of the American Academy of Pediatrics to train large numbers of birth attendants worldwide in basic resuscitation and immediate newborn care [8]. HBB was launched in 2009 in Tanzania and globally during 2010 [9]. The first module in the HMS series, Helping Mothers Survive Bleeding After Birth (HMS BAB) was developed by Jhpiego and partners to train birth attendants in prevention and basic management of postpartum haemorrhage [10]. Subsequently, Essential Care for Every Baby and Essential Care for Small Babies have been added to the HBS suite of educational programs to address the three major causes of neonatal death. Additional modules on management of preterm birth and hypertensive disorders of pregnancy have been added to the HMS series with normal labour and complications of labour under development.

#### 1.1.1 Educational efficiency—Knowledge and skills translation to caregivers

Educational field testing as part of the formative evaluation of HBS programs and HMS BAB suggested high educational efficacy as measured by learner satisfaction, confidence, and gains in knowledge and skills [8,10,11]. Evaluations of the educational effectiveness of HBB have been conducted in Rwanda, Ethiopia, and Honduras demonstrating increased knowledge and skills scores and learner satisfaction [12–14]. However, after a single training in HBB most participants could not demonstrate perfect performance of all steps in bag-mask ventilation [8,12]. HMS BAB training in rural Tanzania, mainly involving midwives, led to an immediate increase in knowledge, skills, and confidence [15], but there followed a decrease in knowledge and simulated basic delivery skills at nine months. However, confidence and simulated obstetric emergency skills were largely retained [16]. A recent study from Rwanda, including 11 physicians, showed an immediate (6–14 days post training) improvement in simulated performance of HMS BAB [17]. The higher level of communication, evaluation, and performance during simulation was sustained after two years among eight physicians available for follow-up testing [17]. In a recently concluded study in Uganda covering 125 facilities in 12 districts where HMS BAB and HBB were combined with ongoing practice after training, performance was sustained particularly by providers in study arms with support for practice (manuscript in preparation).

#### 1.1.2 Local implementation—Translation to clinical practice

Experience from rural Tanzania provided additional confirmation that a one-day training in HBB resulted in improved simulated performance of routine care and newborn resuscitation; however, this improvement was not reflected in the clinical management of actual births as recorded by independent observers [18]. Implementation of systematic low-dose high-frequency training in the labour ward led to changes in clinical management with reproducible reductions in first day mortality [19]. Audits, case reviews and some debriefing with self-reflection were also performed in the same period [19].

A larger Tanzanian implementation trial took place in 8 hospitals with a total of 8,124 births before and 78,500 after HBB training and continued refresher practice. Neonatal deaths at 24 hours decreased by 47% and fresh stillbirths decreased by 24% [20]. Implementation follow-up from Karnataka, India (4187 births before and 5411 after training) also showed a 46% reduction in fresh stillbirth with no change in predischarge neonatal mortality [21]. Thus, it became apparent that HBB could impact both neonatal death and misclassified fresh stillbirth; however, practice and deliberate efforts to support new patterns of clinical care were necessary to achieve these reductions in mortality.

In a tertiary maternity hospital in Nepal, HBB was implemented with quality improvement process cycles to reinforce resuscitation procedures [22]. The intrapartum stillbirth rate decreased from 9 to 3.2 per thousand deliveries, and first-day mortality from 5.2 to 1.9 per thousand live births. After intervention, the odds of inappropriate use of suction and stimulation decreased by 87% and 62%, respectively. Prior to intervention, no babies received bag-mask ventilation within 1 minute of birth, compared to 84% of babies after [22]. A large cluster-randomized trial of HBB, including 52 clusters in semi-urban and rural sites in India and Kenya, concluded that a rapid scale up of HBB training of facility birth attendants was associated with reduced perinatal mortality and fresh stillbirth at Kenyan facilities where training occurred and improved survival of low birthweight infants in India. However, there was no improvement in population-based perinatal mortality (fresh stillbirths and deaths in the first 24 hours among births occurring both in and out of facilities) [23]. Additional implementation trials are underway employing a variety of approaches to continue training practice and deliberately improve skills and clinical management. Ongoing studies provide important insights on how health systems can implement HBB to address their unique situations and achieve sustained results.

Critical examination of the experience and lessons learned in 73 countries during the first 5 years of implementation of HBS highlighted the need to focus on implementation [24]. In order to achieve dense coverage with the educational interventions and utilize the programs as catalysts for improvement in the quality of maternal and newborn care, local implementation by the bedside provider becomes paramount. A convocation of representatives from implementing groups was organized as an Utstein consensus conference to make recommendations on the key steps in implementation of HBS/HMS programs to increase the number of maternal and newborn lives saved.

### 1.2 Utstein formula for maternal and newborn survival

**Fig 1:**
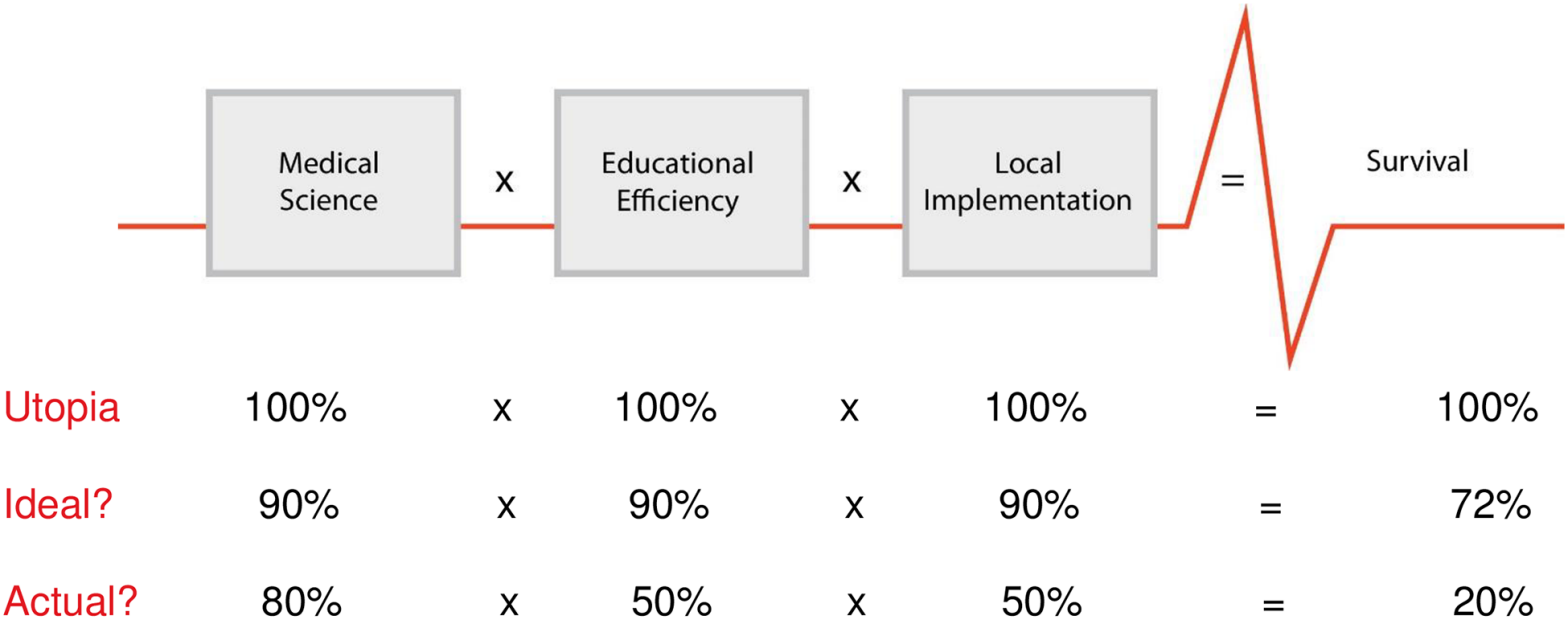
The “Utstein Formula for Survival” with different implementation scenarios (adapted from Søreide et al [26] with permission).

In 2003, the International Liaison Committee on Resuscitation (ILCOR) published an Advisory Statement on Education and Resuscitation, conceptualizing a hypothetical “formula of survival” (Fig 1) [25]. The formula was developed during a resuscitation education symposium in the Utstein Abbey, Stavanger, Norway in 2001. The “formula for survival” materialised in relation to cardiac arrest patients, and stipulates that patient outcome is a product of three interactive factors; medical science (guideline quality), educational efficiency (knowledge translation to patient caregivers), and local implementation (actual delivery of health care). The validity of this hypothesis and the influence of each factor on survival were discussed at another Utstein meeting in 2006 [26]. It is predicted that all factors in the formula contribute equally to patient outcome. Hypothetically, if all the factors are optimal (1 x 1 x 1 = 1), patient survival would be 100% [26]. In the real world, gaps exist in medical guidelines, but even more, educational efficiency limits diffusion of best practices and local implementation constrains uptake. Improvement in one or all three multiplicands will improve patient outcome. Although defining a precise value of each factor may be difficult, the “formula for survival” provides a conceptual framework and can be used as a template to improve health care systems related to many different patient groups and to understand variability in patient outcome among sites and countries.

Historically, health research has focused on medical science and educational methods. Relatively little attention has been directed towards scaling up educational programs or the aspects of educational efficiency which translate acquired skills into clinical practice and patient impact. Limitations in policy, administration, physical environment, finances, technology, staffing, supply and logistics all pose challenges at the level of local implementation. Therefore, the local implementation factor is probably the weakest multiplicand of the formula in most settings throughout the world.

Using the Utstein process, we focused on the second and third elements, the multiplicands of educational efficiency and local implementation. We sought to identify key steps in dissemination of HBS/HMS educational programs and application of the knowledge and skills to the delivery of clinical care which would increase maternal and newborn survival.

### 1.3 The objectives of this Utstein process are

1. To provide “on the ground” users of HBS and HMS programs a minimum list of key steps/actions to create sustained impact, leading to increased survival of babies and mothers
2. To disseminate the discussions/conclusions of the process in order to inform both policy and program leaders as well as health workers and their communities

## 2. Materials and methods

### 2.1 The Utstein process

Since the first Utstein meeting in 1991, 25 Utstein-style meetings have been conducted to reach international consensus on topics related to resuscitation and resuscitation research: how to measure and report survival data in out-of-hospital cardiac arrest, in-hospital cardiac arrest, pediatric emergencies, trauma, and laboratory resuscitation research [27,28]. Altogether 16 consensus papers have been issued. Prior to the meetings, participants are provided with background information summarizing the areas for discussion, the state of knowledge around those topics, and the challenges and barriers encountered. Participants are asked to reflect on their experience and prepare to contribute successful strategies for discussion and broader debate during the proceedings. The Utstein consensus process consists of a series of rotations of participants through a set of topics with the goals of first obtaining broad input and then successively refining conclusions and recommendations. In the first rotation, a moderator outlines the scope of the topic and elicits input and discussion from participants; the moderator and a recorder capture the discussion. In subsequent rotations, the moderator summarizes previous points and moves the discussion toward prioritizing key points and recommendations. All topics are presented for comment and amendment during plenary sessions and distributed in written form for final comments from the entire group.

Participants in the consensus conference represented diverse global implementing partners of the HBS/HMS educational programs, including those unified under the umbrella of Global Development Alliances (GDAs). Founding partners of the Helping Babies Breathe GDA included the United States Agency for International Development (USAID), the American Academy of Pediatrics (AAP), Save the Children/Saving Newborn Lives, Laerdal Global Health, and the Eunice Kennedy Shriver National Institute for Child Health and Human Development (NICHD). In 2012, the HBB GDA expanded to the Survive and Thrive GDA with the inclusion of The American College of Obstetricians and Gynecologists (ACOG), American College of Nurse-Midwives (ACNM), Jhpiego, Johnson & Johnson, and subsequently, more than a dozen additional partners. Participants in the consensus conference were drawn from the founding GDA partners, implementing partners of USAID (Jhpiego, MCSP, URC, PATH), Ministries of Health (Tanzania) and universities in implementing countries, UN health agencies (UNICEF, UNFPA), foundations (Bill and Melinda Gates, CIFF, ELMA, NIPI), and professional organizations (FIGO, ICM, AAP, ACNM, ACOG, AHA, Indian Academy of Pediatrics, Ethiopian Pediatric Society).

### 2.2 Data collection

#### 2.2.1 Pre-meeting inputs

In advance of the meeting, all invited participants were requested to describe 3–5 key steps in implementation of HBS/HMS programs that increase the survival of mothers and babies. Participants received an electronic copy of a journal article describing the Utstein process and also an electronic copy of the HBB Global Development Alliance 5-year report; *Helping Babies Breathe*: *Lessons learned guiding the way forward* [24].

Responses were analyzed according to *a priori* and emergent codes to identify central themes (Table 1). Development of *a priori* codes took place prior to compilation of the pre-meeting inputs. Codes globally represented domains of the RE-AIM evaluation framework for health interventions (reach, effectiveness, adoption, implementation, maintenance) and incorporated specific issues identified (lessons learned) from the report *Helping Babies Breathe*: *Lessons learned guiding the way forward* [24]. Emergent codes were reserved for new themes not fitting under any *a priori* code or bridging several codes.

**Table 1:** A priori and emergent codes based on the pre-meeting inputs.

**A priori codes for thematic analysis**–including subordinate themes	
**A**	Work with stakeholders who are ready for implementation–nationally, in-facility
**B**	Include all stakeholders–professional associations, UN health agencies, maternal/neonatal, public/private
**C**	Make a national plan including the Ministry of Health–accommodating new inputs/unforeseen changes, realistic and phased
**D**	Designate leadership for carrying out the national plan
**E**	Define the role of stakeholders clearly–site x time, small-scale partnerships, collaboration to avoid compartmentalization
**F**	Have a system for accountability that is transparent to all parties–nationally, in-facility
**G**	Establish a system for training–train-the-trainer-and-provider cascade, fidelity, coverage, adaptation of materials, integration of maternal/neonatal content, video
**H**	Conduct training in-facility–local ownership, prioritized content, high staff coverage
**I**	Conduct low-dose, high-frequency practice–tailored to needs, incentivized, self-evaluation checklists, video
**J**	Identify a local champion in-facility
**K**	Introduce programs (or content) into pre-service curricula
**L**	Support a facility-based improvement process using HBS/HMS outcome/process measures–flexible leadership, various sources of materials, content based on needs
**M**	Build a reliable supply chain/procurement/maintenance/reprocessing system
**N**	Encourage community participation/mobilization–awareness/advocacy, family training for out-of-facility births and essential newborn care
**O**	Collect data on core set of outcomes
**P**	Align national health registry data and facility-based data collection
**Q**	Utilize data for guiding improvement and budgeting
**R**	Enact policies/regulations supporting high-quality care–training, commodities, facilities, personnel
**S**	Budget at all levels to support high-quality care
**Emergent codes**	
**1**	Integration
	a-between Ministry of Health, educational bodies, professional associations and hands-on workforce
	b-between maternal and neonatal domains at all process steps from readiness to funding to planning/stakeholder roles, training, practice (integrated scenarios), data
	c-health facilities and community–prenatal care, in-facility care, community follow-up
	d-regionalized care–how to get advanced help, time to advanced care, public and private facilities
**2**	Empowerment of provider, pregnant woman, family

#### 2.2.2 Structure of the 2-day Utstein-style consensus meeting

**Fig 2:**
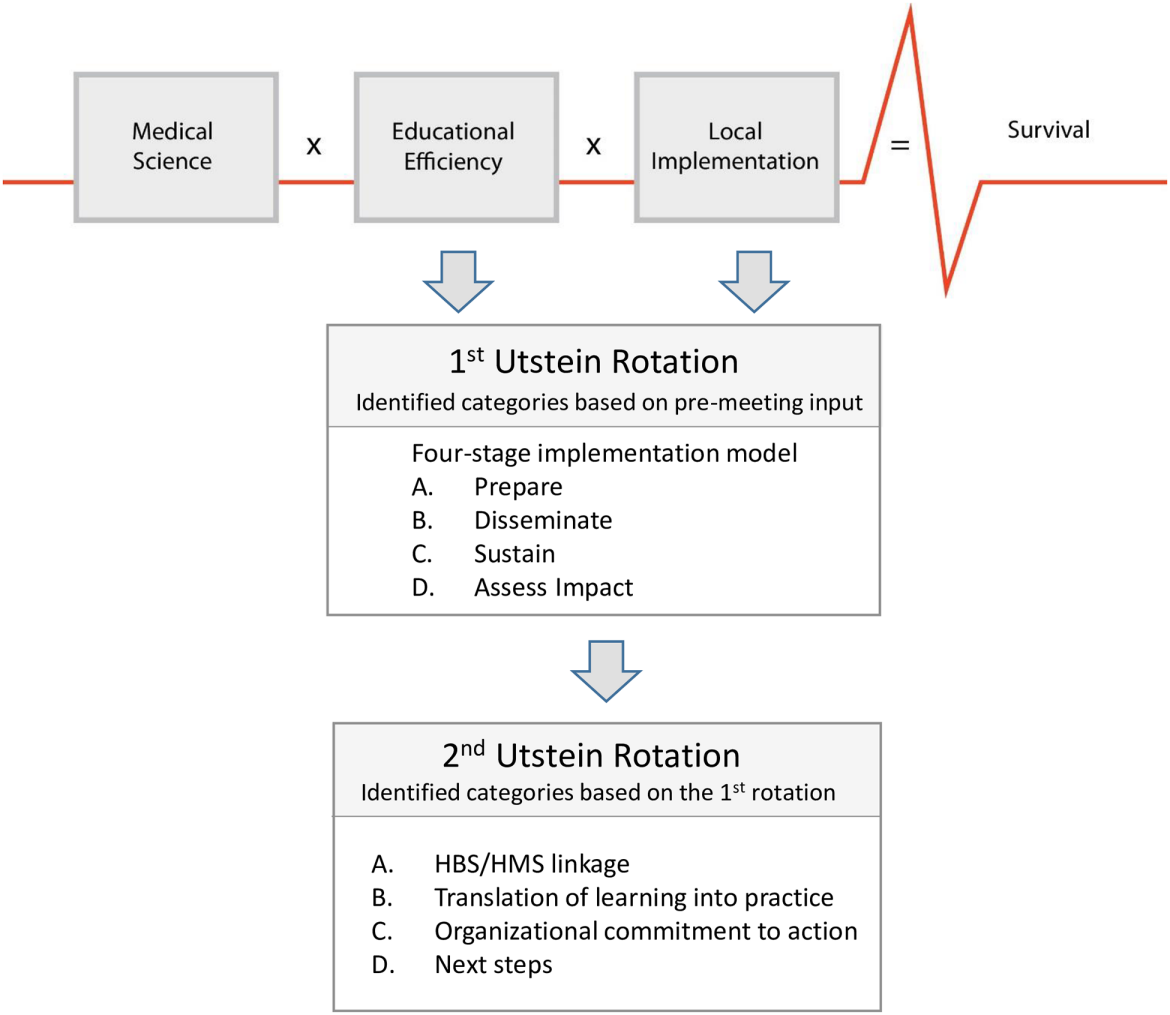
Illustration of the 4-stage implementation model and categories for discussion during the 1^st^ and 2^nd^ Utstein-rotation related to the “Utstein Formula for Survival” (adapted from Søreide et al [26] with permission).

The first Utstein-style rotation focused on four main identified categories (Fig 2):

A. Prepare: partners and strategic planning; B. Disseminate: education and systems for delivery of training; C. Sustain: maintenance and implementation with quality improvement; D. Assess Impact: monitoring, evaluation, and feedback to strategic planning.

The participants were divided into mixed groups rotating among four stations (A, B, C, D) in different sequences. Moderators facilitated the discussions and took notes, as did one participant for each group. Moderators provided the summary of the previous rotations; the first two rotations focussed on the broader picture and the next two on identifying and reaching agreement on the most important issues. Finally, each moderator presented a summary of the outputs to the whole group, stimulating a plenary discussion to confirm consensus.

The second Utstein-style rotating group session focused on identified outputs from the first Utstein-style session and the way forward. The topics for discussion included: A. How can linkages among the HBS/HMS educational programs improve educational efficiency?; B. What facilitates the translation of learning into practice?; C. What can the individuals and organizations of the GDA do to help operationalize the action points?; D. What are the next steps in this Utstein process? (Fig 2)

#### 2.2.3 Post-meeting synthesis of group discussion and operationalization of the key steps

The co-chairs convened and worked with participating local experts from countries with ongoing HBB implementation to refine and illustrate the list of key steps with concrete examples and operational approaches to create sustained impact.

### 2.3 Ethical consideration

No specific ethical clearance was needed to conduct this consensus meeting and review process. Conference participants agreed to represent their organizations specifically to engage in building consensus. In addition, during the consensus meeting, all the participants agreed upon the need for an open-access paper describing the outcomes of the consensus process. By e-mail reply to the corresponding author, all participants approved the final manuscript for submission and confirmed that their names could be published in the acknowledgement.

## 3. Results

### 3.1 Transcription of pre-meeting inputs

More than 20 individuals and groups of participants responded to the request for input. Many suggestions incorporated multiple aspects in a single step.

The most frequently mentioned processes for successful implementation of HBS and HMS included:

1. Work with stakeholders who are ready for implementation (national and facility level)
2. Include all stakeholders in maternal and newborn health (professional associations, public/private, policy/funding/implementing organizations of all types)
3. Make a national plan including the Ministry of Health (leadership, ownership, detailed timeline/phases, contingencies)
4. Establish a system for training (cascade, fidelity, coverage, adaptation, integration of maternal/newborn content)
5. Conduct low-dose, high-frequency practice (tailored to needs, incentivized, self-reflective)
6. Support a facility-based improvement process using HBS/HMS outcome/process measures (based on needs)
7. Build a reliable supply chain/procurement/maintenance/reprocessing system
8. Encourage community participation/mobilization (awareness/advocacy, training of families in basic care)
9. Collect data on a core set of outcomes
10. Utilize data to guide improvement and budgeting
11. Strengthen policies/regulations supporting high-quality care (training, commodities, facilities, personnel)

Integration was a cross-cutting theme mentioned at several levels, including that of organizations (Ministries, educational bodies, professional associations and workforce, public and private facilities), maternal and newborn care (educational content, training, practice), health facilities and the community, primary and referral facilities (regionalization for advanced care). Empowerment of the provider and the woman/family also emerged as themes.

Based on the inputs from the participants the co-chairs developed a four-stage implementation model linked to the “Utstein Formula for Survival” (Fig 2).

### 3.2 Outputs from the Utstein consensus meeting

Forty-eight participants from 22 countries participated in the 2-day meeting in June 2015.

#### 3.2.1 Outputs from the first Utstein rotation

The first Utstein-style rotation focused on the four-stage model (Fig 2). Outputs, meant for “on the ground” users of HBS/HMS programs, were summarized as follows:

A. Prepare—partners and strategic planning

- Define the gap or need in the current system and establish it as a national priority
- Join or create an inclusive working group with the Ministry of Health
- Identify funding partner(s)
- Design national plans for learning phase, roll out, and sustainability

B. Disseminate—education and programmatic planning

- Establish and implement service delivery standards
- Develop a comprehensive (national) training plan
- Build capacity at all levels (women/families, trainers, providers and selected champions)
- Institutionalize program in pre-service education

C. Sustain—implementation with quality improvement

- Form maternal newborn and child health (MNCH) alliance for local levels
- Design programs (low-dose high-frequency) based on national guidelines with inputs from local providers
- Implement regular feedback loops on practice performance (clinical data, impact data, education, equipment) to drive emotional connection and skills perfection where possible
- Generate comparative status reports for maternal/newborn programs
- Identify and support local champions
- Design a system in which users can evaluate care
- Empower birth attendants, pregnant women, and families to improve care

D. Assess Impact—monitoring and feedback

- Collect and utilize data at the point of care delivery; certification of facilities should in part depend on data collection; allow all birth attendants to register births/data
- Define goals/criteria for success
- Involve providers in determining what data to collect and utilize data of different scope at different frequency
- Seize the opportunity to help shape health management information system (HMIS) in country to utilize uniform definitions/datasets
- Advocate for universal civil registrations
- Inform standardized and validated global indicators

#### 3.2.2 Outputs from the second Utstein rotation

The second Utstein-style rotation focused on identified outputs from the first rotation and the way forward (Fig 2):

1. How can we help link the HBS/HMS educational programs to improve educational efficiency?
2. How can we help the care provider translate learning into practice (behavior change)?
3. What can you/your organization do to help operationalize the action points?
4. What are the next steps in this Utstein process?

Outputs of the second rotation included:

A. Linkage HBS/HMS:

- Reinforce ongoing Basic Emergency Obstetric and Neonatal Care (BEmONC) and Skilled Birth Attendant (SBA) training–using short sessions of HMS/HBS training
- Align content of HBS and HMS modules–acknowledge the continuum of care for the mother-infant dyad—Put care for women on HBB Action Plan
- Consider combined Action Plan for basic delivery and wider wall chart of care duties across all modules
- Align approaches across modules for use of models, trainer development, simulation, debriefing, low-dose high-frequency (LDHF) training

B. Translating learning into improved care:

- Practice: vital for maintenance/improvement of skills–opportunities during self-reflection after resuscitation (including video-guided review), patient care handoff, LDHF training, mentorship
- Real-time actionable feedback on quality of resuscitation in practice sessions—need to objectively measure quality of resuscitation practice and outcome and use that data for facility based quality improvement
- Develop mechanisms that get frontline health workers emotionally attached to routine skills practice needs
- Quality improvement: process analysis, case review with systems change, improvement team carrying out specific quality improvement cycles directed by the measured needs

C. Organizational commitment:

- Internal communication/advocacy/consistency–promote formal statements and engage with global partners
- Proactive work with national working groups–support national professional bodies, leadership, planning and transition to pre-service education as the focus of training
- Translate key implementation materials into global languages and support integration of training modules
- Shift from global to national ownership of program/strategy
- Expand/revise Survive and Thrive GDA membership to reflect national voices and visions of health systems technology
- Catalyze global south-south experience sharing among countries/regions implementing HBS/HMS programs

D. Next steps in this Utstein process:

- Participants agreed upon the need to understand and create awareness of implementation factors at national/health system/facility levels in order to make a sustained change in clinical management that will improve survival.
- Co-chairs were asked to convene in-country program leaders with practical HBS/HMS experience and collaborating researchers to illustrate and operationalize the outputs from the Utstein meeting.
- Participants agreed upon the need for a simple document and an open-access paper describing the universal minimum “on-the ground” or in-country steps/factors in preparing and implementing HBS/HMS successfully for use as a tool guiding in-country decision makers and/or program users/implementers

### 3.3 Outputs from the core group discussion/collection of practical country experiences

Ten participants, who had practical experiences implementing HBS/HMS programs in several countries, formed the core group of co-authors and operationalized the consensus conclusions. As examples of different implementation approaches, summaries of implementation processes in Nepal, Tanzania, and Uganda are presented below.

**Fig 3:**
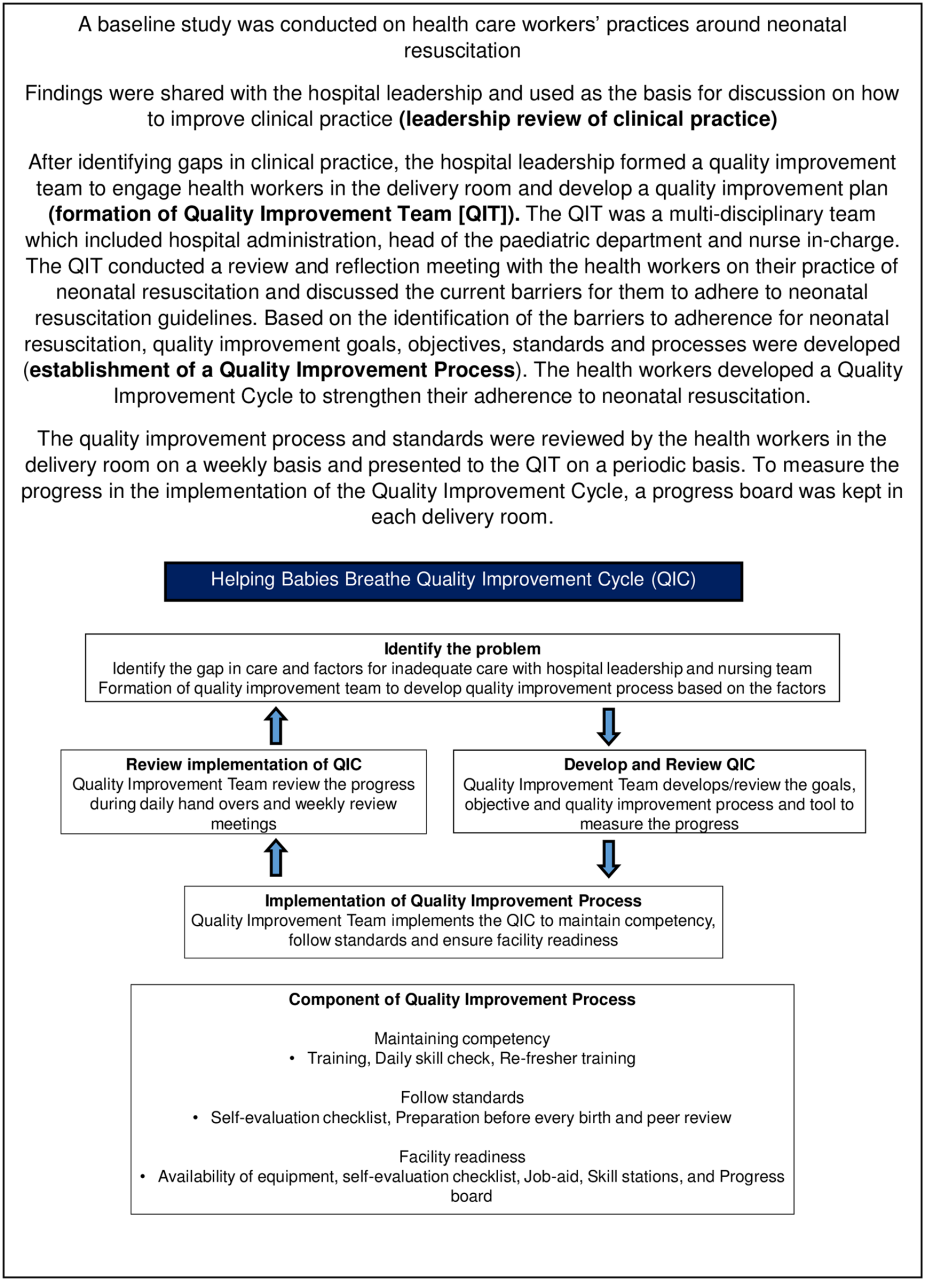
Nepal: Formation and function of the quality improvement team.

In Nepal, a large maternal-infant hospital established a Quality Improvement Team (QIT). The formation and function of the QIT are described in Fig 3. The quality improvement process and standards became routine for the delivery room with support from the QIT. The QIT model is currently being extended to 12 district hospitals, with support of UNICEF Nepal.

**Fig 4:**
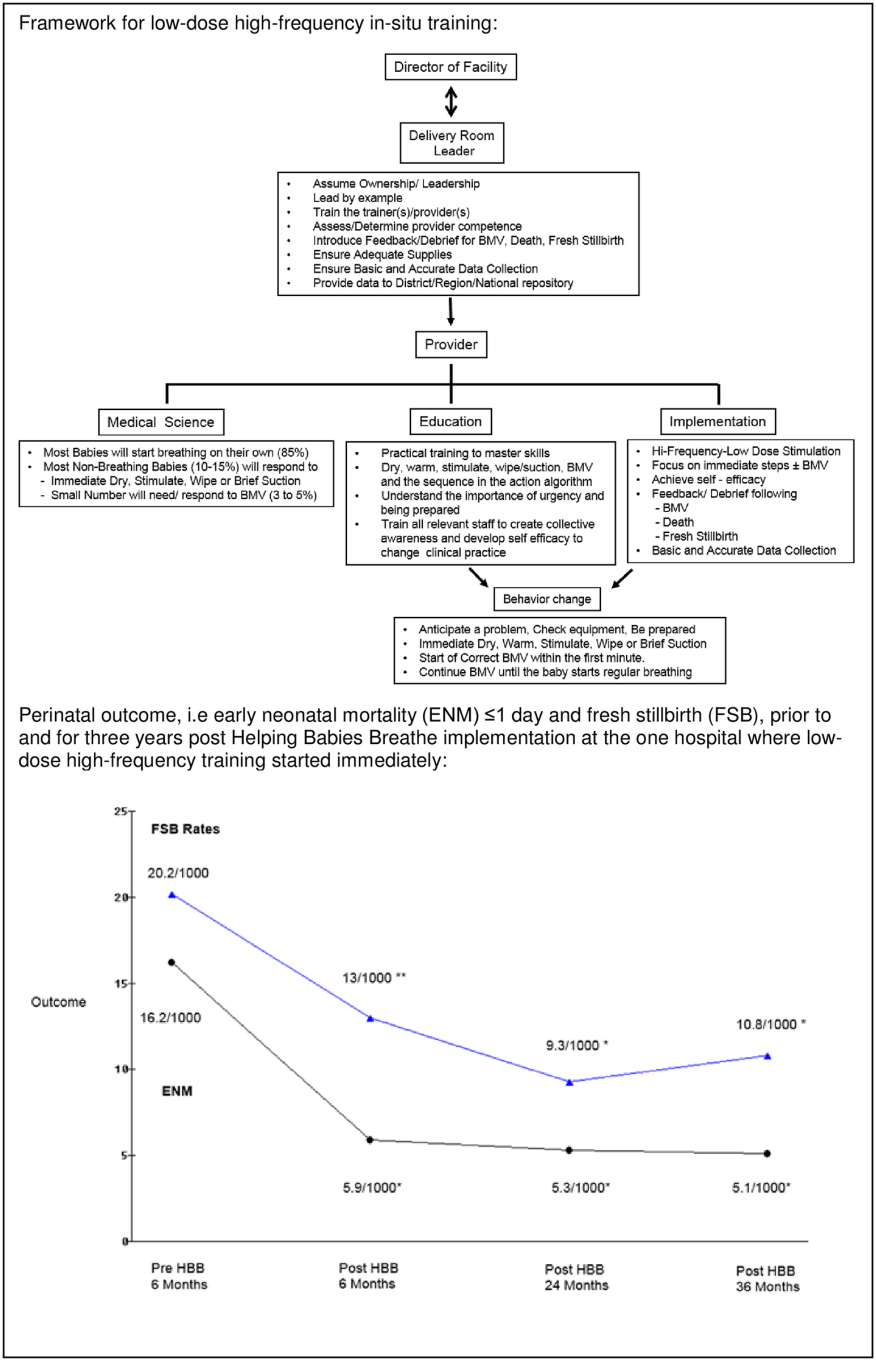
Tanzania: Champions of LDHF in-situ training (adapted from Perlman et al [29] with permission).

In Tanzania, the Ministry of Health led implementation and evaluation of HBB at eight hospitals across the country with financial support from AAP and the Laerdal Foundation. Both the inital and continued success derive from national ownership and commitment, which resulted in complete buy-in and ownership of the head midwives in the labor wards [29]. At one site, development and implementation of LDHF in-situ HBB training started immediately. A manikin (NeoNatalie, Laerdal Global Health) was placed in the labour suite, and it became mandatory for all staff to practice before starting each shift. The framework for LDHF in-situ training and immediate effects (Fig 4) were shared at the bi-annual data monitoring meeting, gathering key midwives from all the sites and stimulating the other sites to start LDHF training. Empowerment of the midviwes in combination with recurrent training are regarded as the keys to sustained impact of HBB in spite of limited resources/financial support.

**Fig 5:**
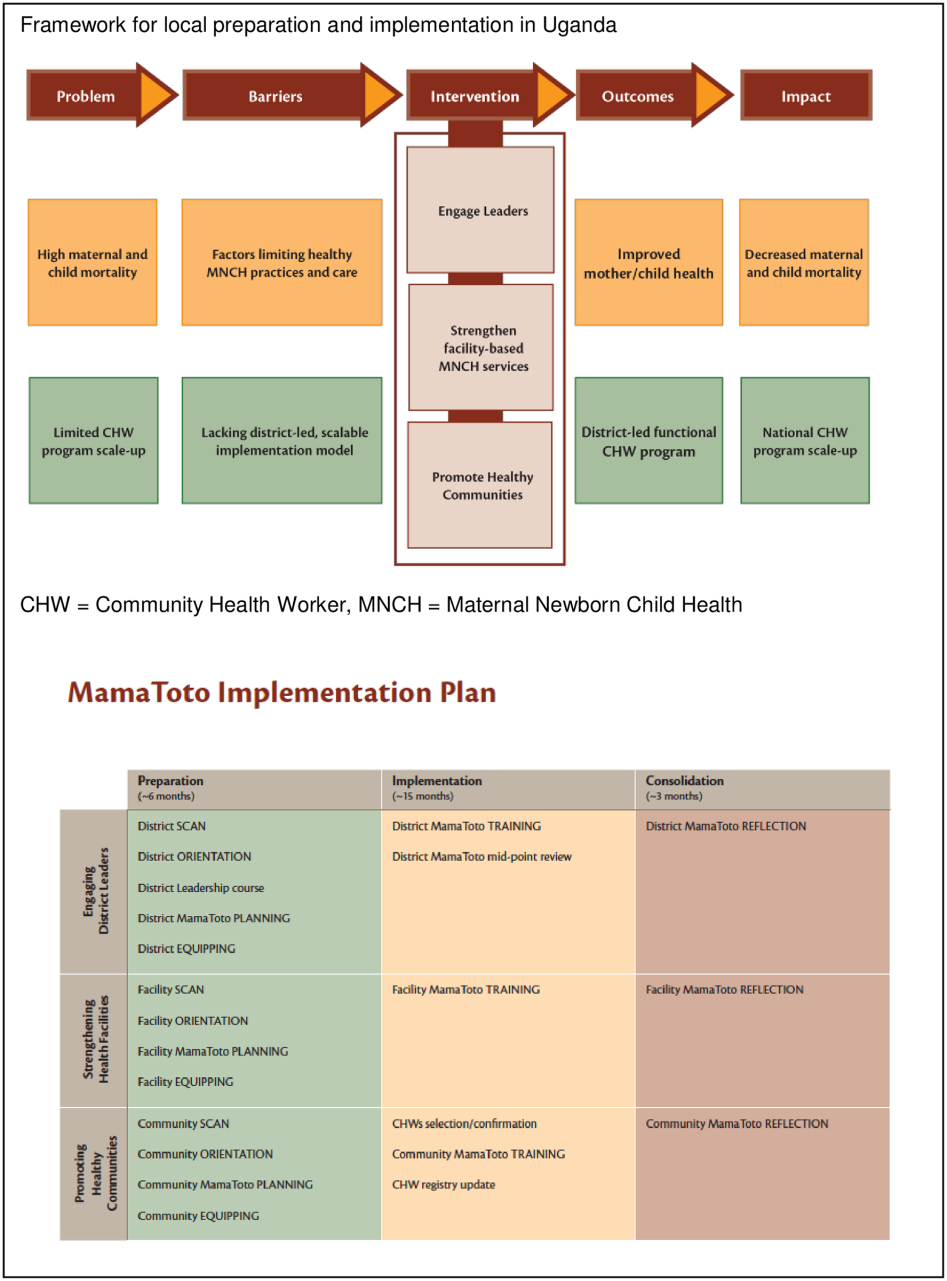
Uganda: Implementing a broader program after scanning and planning.

In Uganda, HBB is implemented as part of a broader “Mama Toto” program to reduce maternal, newborn and child deaths [30]. The Ministry of Health and District Health Authorities are an integral part of the project. Fig 5 depicts the implementation model for this hybrid trial, illustrating that scanning the environment and planning with the partners before delivering any education leads to more sustainable programs, better buy-in from all healthcare providers, and improved outcomes [31].

### 3.4 Ten key actions for implementation of HBS/HMS programs

Based on outputs from the Utstein meeting and discussions in the core group, a list of 10 essential actions points for national HBS/HMS implementation was developed (Table 2).

**Table 2:** Essential action points for national HBS/HMS implementation.

1. At the country level, establish a maternal/newborn/child health alliance with public, private, and non-governmental partners
2. Form a functional working group for advocacy, planning, training, and monitoring at the country level. Through the working group, identify gaps in the current system, establish performance standards, set specific goals, and develop a financial plan to implement and sustain the program(s)
3. Develop a plan for national-to-facility levels training, which achieves high-quality coverage of providers in both public and private facilities
4. Provide appropriately adapted learning materials, equipment and supplies simultaneously with training
5. Identify and support local leaders and champions
6. Set up local systems for frequent, brief refresher training, debriefing, and audits
7. Support the function of facility-level perinatal quality improvement teams
8. Collect and report local data on a standardized set of indicators of basic processes of care and patient outcomes
9. Develop a system for looped reporting and feedback to/from all levels of the health system and the working group
10. Engage and empower health care providers, families, and the broader community in the initiative

## 4. Discussion

The Utstein-style consensus process, engaging a broad international group of experts, produced a 10-point list of essential HBS/HMS implementation action steps. These steps are designed to promote successful national implementation of HBS/HMS, and should be useful at many levels, from country policy makers to “on the ground” frontline health workers, as well as international organisations/stakeholders.

The HBS/HMS Utstein meeting marked the extension of the Utstein consensus process into consideration of the third element in the formula for survival–implementation. Previous Utstein meetings have focused mainly on medical science (resuscitation research, measuring performance and uniform reporting of resuscitation data), the first multiplicand in the Utstein Formula for Survival (Fig 1). Recognizing the great variance in patient outcomes among facilities and settings, despite similar medical guidelines and education programs, the HBS/HMS Utstein meeting occurred in parallel with another Utstein meeting also focusing on implementation: “Improving Survival from Out-of-hospital Cardiac Arrest–a Call to Establish a Global Resuscitation Alliance” [32]. The main focus of both meetings was to develop an implementation strategy for successful translation of knowledge and skills to clinical practice that could be sustained on a national level, illustrating how educational efficiency and local implementation have generalized applicability to a variety of health issues. Within many fields of medicine, there is a growing realization that optimal care, and thus patient outcomes, are not being achieved, and that effects will remain localized and/or limited unless a successful formula for implementation is developed, replicated and adopted widely.

The HBS and HMS educational programs are built on the most current medical science, the same platform that underlies educational programs for high-resource settings. Field-testing and implementation trials in several countries around the world demonstrate high learner satisfaction, confidence and increased knowledge and skills scores [8,10–18]. However, a single HBB training is probably not enough to master bag-mask ventilation [8,12], and a drop in knowledge and simulated basic delivery skills were noted in rural Tanzania, nine months after a single HMS BAB training [16]. Although educational effectiveness of the HBS and HMS programs is good, studies reveal a need for additional efforts to support the translation into clinical practice necessary to improve patient outcome and equalize great disparity across countries [18–22]. Factors that hinder or facilitate the use of best practices on local and national levels need to be rigorously investigated far more intensely than today. Meanwhile, a consensus recommendation by a group of experts can help define necessary steps, through sharing and analysis of experiences.

To make a change on the ground, international organizations, stakeholders and country leaders have to pay more attention to “how to make things happen and monitor what is really happening”. The reported 10-point list of essential HBS/HMS implementation action steps for “on the ground” users can be used as a concrete means to guide implementation processes in low-resourced countries (Table 2). Prior to implementation of any program there has to be an agreement of the scope, commitment and readiness to follow-up. This may be established through a national maternal/newborn/child health alliance. The successful experience from Tanzania demonstrate the importance of a functional (national) working group to lead and push the implementation process by; 1) creating accountability facilitating local buy-in and establishment of national and local ownership and pride, 2) identifying own gaps and defining appropriate service delivery standards with associated specific patient outcome goals, 3) depending on the available financial resources for dissemination and sustainment. The working group should consider and debate their own challenges and implementation costs in the light of possible associated survival impact.

Another key message from Tanzania and Nepal is the importance of making systems that listen to and focus on the birth attendant–empowering the midwives (Figs 3 and 4). Often the key to successful implementation, on the ground, lies at the local level where resources must be mobilized to handle specific tasks and challenges. Behaviour change can be very difficult, and there are multiple reasons for systemic and personal resistance. Representatives of “on the ground” health care workers understand the dynamic and feel the constraints in the field. Listening to their concerns and being attentive to their advice may be critical especially when developing the plan for the training program and to achieve buy-in. Therefore, inviting actual providers from a diversity of facilities into the working group may help customizing the training program to local systems and available resources.

When rolling-out a new program, providers must comprehend the standards and why they matter. The standards must be reasonable, and the goals achievable within a particular setting. There was a strong consensus in the Utstein process that national standards and goals had to be defined and anchored within the country to best reflect the real situation and thereby create ownership and commitment throughout the country. The adapted learning materials should communicate the set standards and goals to all participants before starting the actual training. Appropriate equipment and supplies should be distributed simultaneously to ensure the possibility to meet the set standards in clinical practice.

As countries move toward the 2030 goals set forth in the Every Newborn Action Plan and Strategies toward Ending Preventable Maternal Mortality, using the framework outlined in the WHO Standards for improving the quality of maternal and newborn care in facilities, robust implementation of HBS/HMS programs can help save the lives of more women and babies.
